# Making *in vitro* conditions more reflective of *in vivo* conditions for research on the teleost gastrointestinal tract

**DOI:** 10.1242/jeb.246440

**Published:** 2024-10-11

**Authors:** Carol Bucking, Nic R. Bury, Henrik Sundh, Chris M. Wood

**Affiliations:** ^1^ Department of Biology, Farquharson Life Science Building, York University, Toronto, ON, M3J 1P3, Canada; ^2^ School of Ocean and Earth Sciences, University of Southampton, National Oceanographic Centre, Waterfront Campus, Southampton, Hampshire, SO14 3ZH, UK; ^3^ Department of Biological & Environmental Sciences, University of Gothenburg, Medicinaregatan 7 B, 41390 Göteborg, Sweden; ^4^ Department of Zoology, University of British Columbia, 6270 University Blvd, Vancouver, BC, V6T1Z4, Canada

**Keywords:** Chyme, Stomach, Intestine, Gut microbiome, Mucosal chemistry

## Abstract

To date, the majority of *in vitro* or *ex vivo* fish gastrointestinal research has been conducted under unrealistic conditions. In a living fish, ionic conditions, as well as levels of ammonia, pH, HCO_3_^−^ and *P*_CO_2__ differ considerably between the different regions of the gastrointestinal tract. These factors also differ from those of the saline often used in gut research. Furthermore, the oxygen gradient from the serosa to the gut lumen is rarely considered: in contrast to the serosa, the lumen is a hypoxic/anoxic environment. In addition, the gut microbiome plays a significant role in gut physiology, increasing the complexity of the *in vivo* gut, but replicating the microbial community for *in vitro* studies is exceptionally difficult. However, there are ways in which we can begin to overcome these challenges. Firstly, the luminal chemistry and *P*_O_2__ in each gut compartment must be carefully considered. Secondly, although microbiological culture techniques are improving, we must learn how to maintain the microbiome diversity seen *in vivo*. Finally, for *ex vivo* studies, developing mucosal (luminal) solutions that more closely mimic the *in vivo* conditions will better replicate physiological processes. Within the field of mammalian gut physiology, great advances in ‘gut-on-chip’ devices are providing the tools to better replicate *in vivo* conditions; adopting and adapting this technology may assist in fish gut research initiatives. This Commentary aims to make fish gut physiologists aware of the various issues in replicating the *in vivo* conditions and identifies solutions as well as those areas that require further improvement.

## Teaser

**Summary:** *In vitro* gastrointestinal research on fish requires better replication of *in vivo* conditions in terms of ammonia, HCO_3_^−^ and ion concentrations, as well as pH, *P*_CO_2__, *P*_O_2__ and microbial community.

## Introduction

*In vitro* gut models have been readily incorporated into mammalian and medical research. Studies using these models, often under symmetrical conditions (i.e. where the same medium bathes both luminal and serosal surfaces), have provided insights into nutrient/drug uptake and factors affecting intestinal epithelial integrity, as well as a better understanding of disease aetiology and prevention (Wang et al., 2023; Kim and Sung, 2024; McCoy et al., 2024). The success of these models has occurred despite knowledge that the gut is not an isolated organ; its function is regulated by the paracrine and endocrine systems, the diet, infections and commensal microbiota. The adoption of *in vitro* techniques for comparative physiological studies of the gut outside the medical research sphere has been less enthusiastic. There is an obvious reason for this: medical research focuses on one species, *Homo sapiens*; in contrast, comparative physiologists are interested in studying the differences among many species. In addition, within the ∼30,000 species of teleost fish, there is much variation: some are herbivores, piscivores, omnivores, planktivores or carnivores; gut function differs between freshwater and seawater environments; some species are stomachless; some have extensive pyloric caecae whereas others do not; the intestine may, or may not, be split into three sections (hind, mid and anterior regions) of varying length. One fish model will not adequately cover these diverse features.

**Glossary**

**Alkaline tide**

This occurs after a meal when the cells of the stomach secrete HCO_3_^−^ into the blood, causing a transient rise in blood pH.

**Chyme**

Fluid consisting of gastric juices and partially digested food that passes from the stomach and along the various sections of the intestine.

**Culturomics**

High-throughput cell culture of gut microbes.

**Euryhaline**

Euryhaline refers to an organism that can tolerate a wide range of salinities.

**Gut-associated lymphoid system**

A collection of tissues involved in the immune response to specific gut microbes and antigens.

**Gut sacs**

A technique for measuring properties of the gastrointestinal (GI) tract using segments of the GI removed from the organism. The section of gut sac can either remain in the same orientation or be everted, and then sealed at one end with suture silk and filled at the other end with an indwelling cannula. The sac can be filled with luminal or serosal fluid, and then sealed and placed in a bath of luminal or serosal media, depending on its orientation.

**Henderson–Hasselbalch equation**

An equation used to calculate the ratio of the concentrations of an acid (A) and its conjugate base at the pH of a chemical solution using the acid dissociation constant, *K*_a_: pH=p*K*_a_+log_10_([A^−^]/[HA]).

**3Rs (Replacement, Reduction and Refinement)**

A framework based on the concepts from *The Principles of Humane Experimental Technique* by W. M. S. Russell and R. L. Burch (1959) to improve animal welfare in research.

**Solvent drag**

A process by which a solvent, such as water, moves across an epithelium by osmosis, taking solutes with it.

**Stenohaline**

Stenohaline refers to an organism unable to withstand wide variation in salinity.

**Two-compartment *in vitro* cell culture model**

The culture of cells on a permeable support to generate an *in vitro* epithelium that separates the luminal (apical) from the serosal (basolateral) compartments.

**Ussing chamber**

An apparatus where either the natural epithelium or a monolayer of cells grown on permeable supports is mounted between two chambers for the measurement of electrical impedance, transepithelial potential and the transport of ions and other molecules across the epithelial membrane.

**Fig. 1.**
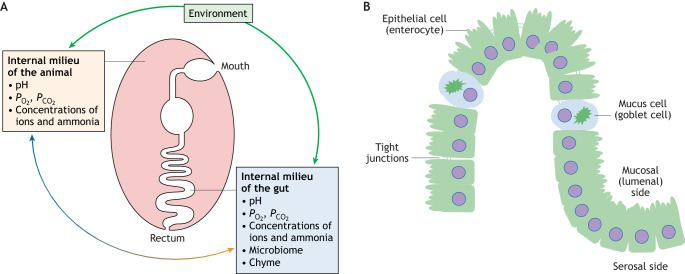
**The fish gastrointestinal tract.** (A) Food and water enter the mouth, and waste food and metabolites exit the anus via the rectum. The gut lumen pH, *P*_O_2__ and *P*_CO_2__, as well as the concentration of ions and ammonia, and the microbiome will vary along the gastrointestinal (GI) tract and will differ between species depending on the environment they inhabit and the food resources available. The gradients of *P*_O_2__, *P*_CO_2__ and ammonia concentration between the gut lumen and the body are influenced by the animal's internal milieu. (B) Schematic diagram of the main cells present in the gut lumen.

There is a societal and political desire to reduce and replace the use of animals in research. However, for comparative research on gut function, it is difficult to see how it will be possible to mimic whole-organism physiology in a flask for multiple species. Thus, *ex vivo* approaches, such as gut sacs (see Glossary) or the use of Ussing chambers (see Glossary) containing gut epithelia from specific species have been used (Weinrauch et al., 2021). Where *in vitro* models are likely to be particularly useful is in research associated with financially important aquaculture species. In fact, *in vitro* technologies have already been developed for salmonids (Drieschner, et al., 2019a,b). However, most of the published work using *in vitro* systems has made use of culture conditions that do not replicate the situation *in vivo*; thus, our Commentary provides details of the challenges in devising *in vitro* systems that consider variations in luminal chemistry, as well as the presence of commensal microbes and anoxia (Fig. 1A). We focus specifically on the teleost gastrointestinal (GI) tract (see Fig. 1B for an overview of the main cell types present).

## Chemistry of the gut lumen

The fluids in the GI tract are part of the external environment from which required substances (e.g. ions and nutrients) are absorbed, and into which waste products may be secreted. Therefore, although it is appropriate in *in vitro* or *ex vivo* work to use physiological saline (a solution designed to mimic the internal environment, i.e. the blood plasma) on the serosal side of the tract, it is not appropriate to use this same solution to mimic the often extreme external environment on the mucosal (i.e. luminal) side. Unfortunately, these are the conditions that have been used in the vast majority of *in vitro* fish studies on gut function.

There is a growing body of literature on the *in vivo* physicochemical conditions of the GI fluids or chyme (see Glossary) in a variety of animals. Here, we consider the freshwater rainbow trout (*Oncorhynvhus mykiss*) as an illustration of what is known regarding *in vivo* conditions in the fish gut, because of its importance as a model organism in physiology, aquaculture and toxicology (Wood and Bucking, 2011). In research on rainbow trout, Cortland saline (Wolf, 1963) is the solution that has been used most frequently as the basis of the mucosal medium for *in vitro* experiments. Usually, it is pre-equilibrated with air, 0.3% CO_2_ (2.3 Torr; where 1 Torr≈133 Pa) or 1% CO_2_ (7.5 Torr); the latter two CO_2_ mixtures may be in air (*P*_O_2__=150–160 Torr) or oxygen (*P*_O_2__>700 Torr). Sometimes, the [Ca^2+^] and [HCO_3_^−^] are reduced to prevent their precipitation. Table 1 compares the composition of standard Cortland saline with measured physicochemical parameters in the luminal fluids in the stomach, anterior, mid and posterior intestine of fasted trout and those fed recently with commercial trout pellets. It should be noted that fish referred to as ‘fed’ in the literature generally receive commercial pellets that do not reflect a natural food source.

**Table 1.**
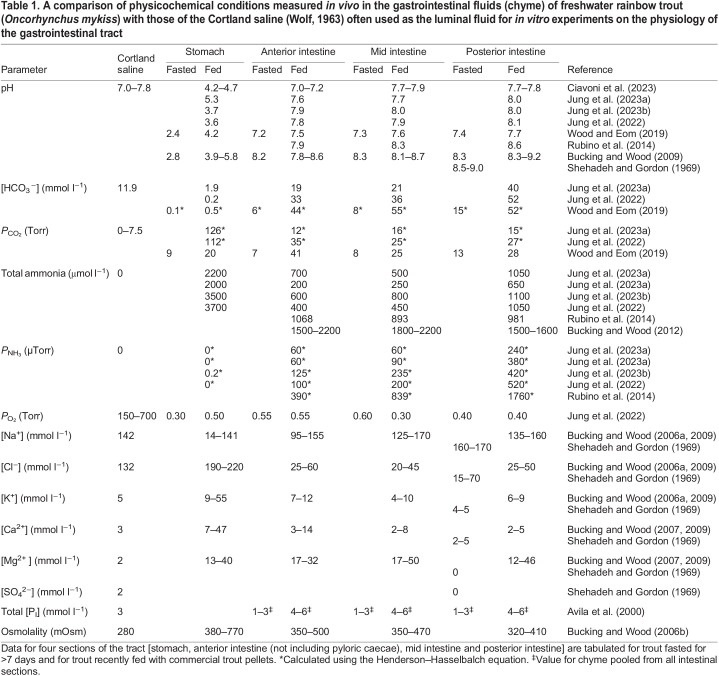
**A comparison of physicochemical conditions measured *in vivo* in the gastrointestinal fluids (chyme) of freshwater rainbow trout (*Oncorhynchus mykiss*) with those of the Cortland saline (** **Wolf, 1963** **) often used as the luminal fluid for *in vitro* experiments on the physiology of the gastrointestinal tract**

### pH

The trout, like many vertebrates (Kong and Singh, 2008) and most but not all fishes (Bakke et al., 2011; Wood, 2019), maintains a very acidic pH in the luminal fluid of the stomach while fasting. The pH rises considerably after feeding as a result of the buffering action of the ingested food (Table 1, and references therein). This occurs despite a massive activation of HCl secretion by the gastric H^+^,K^+^-ATPase, which creates a reciprocal alkaline tide (see Glossary) in the blood (Bucking and Wood, 2008; Cooper and Wilson, 2008), both of which can persist for more than a day (depending on the species) after feeding. The Cortland saline commonly used *in vitro* has a pH of 7.0–7.8, which is 2–5 pH units above the real *in vivo* pH for the gastric epithelium, which has a 100- to 10,000-fold greater H^+^ concentration bathing its mucosal surface. In freshwater fish, the stomach has important roles in the absorption of major ions (Bucking and Wood, 2006a, 2007, 2009), ammonia (Jung et al., 2023b) and nutrient metals such as nickel (Leonard et al., 2009) and copper (Nadella et al., 2011). The availability and ion speciation of these are affected by the low gastric pH. Similarly, low pH is essential for proper activity of gastric proteases (Márquez et al., 2012). *In vitro*, low pH in the luminal fluid can be maintained using buffers such as formic acid (Jung et al., 2003b) or PIPES (Nadella et al., 2011) (the latter of which does not complex metal ions; Kandegedara and Rorabacher, 1999).

In contrast to the stomach, all sections of the trout intestine are alkaline, with pH increasing progressively from the anterior to posterior sections, and greater alkalisation after feeding (see Table 1, and references therein). The pH of the chyme in the posterior intestine may approach 9.0 in fed trout; again, well outside that of the Cortland saline, but in the opposite direction from that of stomach chyme. In trout and other fish, the cause of the high pH is HCO_3_^−^ secretion by the mucosal epithelium (Wood, 2019). It is well established that the optimal activities of digestive enzymes are very sensitive to luminal fluid pH in salmonids (Krogdahl et al., 2015), as are the intestinal uptake rates of copper (Nadella et al., 2007) and iron ions (Kwong and Niyogi, 2008). It is critical that realistically high pH values are applied on the mucosal surfaces of *in vitro* preparations. Although this can be accomplished using buffers that do not complex metals (Kandegedara and Rorabacher, 1999), it is best done by appropriate manipulations of *P*_CO_2__ and [HCO_3_^−^] (using the Henderson–Hasselbalch equation; see Glossary; Boutilier et al., 1984) to properly mimic the *in vivo* situation.

### HCO_3_^−^ concentration

[HCO_3_^−^] is negligible in gastric fluids, as a result of the low pH (Table 1). However, the presence of high [HCO_3_^−^] in the intestinal fluids as well as the reciprocal variation of [HCO_3_^−^] with [Cl^−^] in the intestine have long been known in marine fish (Smith, 1930), including the seawater-acclimated rainbow trout (Wilson et al., 1996). In seawater, Cl^−^/ HCO_3_^−^ exchange serves to absorb Cl^−^ and secrete HCO_3_^−^; this results in the precipitation of divalent cations (Ca^2+^ and Mg^2+^) and HCO_3_^−^ as carbonates out of solution from the ingested seawater (Walsh et al., 1991), thereby improving the osmotic gradient for water absorption from the lumen into the blood (Grosell, 2006; Wood, 2019). However, only recently has it been shown that luminal [HCO_3_^−^] may also be very high in the freshwater-acclimated rainbow trout. The values tend to increase progressively from the anterior to posterior intestine and are especially high after feeding, sometimes exceeding 50 mmol l^−1^ (Table 1). This is true not only in the trout (a euryhaline teleost; see Glossary) but also in several stenohaline freshwater teleosts (see Glossary; Wood and Eom, 2019; Goodrich et al., 2020). There is no obvious role for this high intestinal [HCO_3_^−^] in osmoregulation. Rather, the high [HCO_3_^−^] balances the high *P*_CO_2__ in the GI tract, thereby ensuring an alkaline pH and setting the correct acid–base conditions for enzyme and transporter functions. In conclusion, HCO_3_^−^ levels in standard Cortland saline are far too high for the mucosal solution of the stomach, and too low for the mucosal solution of the intestinal segments, at least after feeding. They should therefore be adjusted accordingly for *in vitro* experiments.

### *P*_CO_2__

In fish, simple calculations using the Henderson–Hasselbalch equation and measured values of pH and [HCO_3_^−^] have demonstrated that *P*_CO_2__ levels in the GI fluids of fish are usually much higher than the 0–7.5 Torr routinely used for Cortland saline (Wood, 2019) (Table 1). This discrepancy increases after feeding and occurs throughout the tract. In fact, *P*_CO_2__ levels in the GI tract may exceed 50 Torr (10–20 times normal arterial blood levels of 2–4 Torr). This has been confirmed by direct measurements of *P*_CO_2__ in luminal fluids with micro-optodes both in freshwater rainbow trout (Wood and Eom, 2019) and in marine English sole (Jung et al., 2020). The high *P*_CO_2__ levels in the chyme result in the diffusion of CO_2_ into the enterocytes, cells that are rich in carbonic anhydrase. Subsequently, CO_2_ is converted to H^+^ and HCO_3_^−^, thereby ‘refuelling’ the supply of these acid–base equivalents, which are vital for ion, metal and nutrient transport processes, as well as for maintenance of luminal fluid pH (Wood, 2019). Undoubtedly, both the enterocytes and the microbes in the GI tract are adapted to life under high *P*_CO_2__. If these conditions are not met at the mucosal surface in *in vitro* preparations, the cells are functioning in a very unnatural environment.

### Ammonia

Ammonia is simultaneously a respiratory gas (NH_3_), an ion (NH_4_^+^), a valuable resource for protein production through the glutamine synthesis pathway and a highly toxic nitrogenous waste. A large amount of ammonia is produced by digestive processes, enterocyte metabolism and microbial activity in the GI tract. This accounts for up to 47% of whole-body ammonia production in the post-prandial rainbow trout, and much of this ammonia is absorbed across the tract (Rubino et al., 2014). In trout, total ammonia concentration in gastric and posterior intestinal chyme may exceed 3500 and 1500 µmol l^−1^, respectively (see Table 1, and references therein). By contrast, blood plasma levels of ammonia are generally less than 200 µmol l^−1^, and Cortland saline lacks ammonia entirely. The partitioning of ammonia between NH_4_^+^ and the more toxic NH_3_ is a function of pH (Cameron and Heisler, 1983), with the NH_3_ fraction (and therefore *P*_NH_3__) increasing as pH increases. Therefore, in trout, *P*_NH_3__ is essentially zero in gastric chyme and highest in posterior intestinal chyme, where pH is highest (Table 1). As with *P*_CO_2__, the enterocytes and microbes are likely to be adapted to these high *P*_NH_3__ levels and total ammonia concentration *in vivo*; ideally, these conditions should be replicated in the mucosal media during *in vitro* experiments.

### Major ions and osmolality

In designing mucosal solutions for *in vitro* experiments, it is critical to know the ionic composition of the normal diet to which the animal is adapted, and how this composition changes in the fluid phase of the chyme as it is sequentially processed along the GI tract. For freshwater rainbow trout fed commercial pellets, [Na^+^] throughout the tract remains generally similar to or slightly higher than levels in Cortland saline or plasma (see Table 1, and references therein). However, [Cl^−^] in gastric chyme is much higher than that of plasma or saline because of HCl secretion in the stomach. [Cl^−^] then drops to much lower levels throughout the intestine as a result of Cl^−^/ HCO_3_^−^ exchange in the intestine (Table 1). Concentrations of K^+^, Ca^2+^ and Mg^2+^ are also elevated in gastric fluid relative to the blood plasma, but unlike [Cl^−^], the concentration of these ions remains generally elevated in luminal fluids throughout the intestinal segments; the elevation in concentration is particularly pronounced for Mg^2+^, for which concentrations may be 10- to 25-fold higher than in plasma or saline. Interestingly, seawater-acclimated rainbow trout fed a diet of commercial pellets exhibit rather similar ranges of major ion concentrations in their GI fluids (Bucking et al., 2011) to those of freshwater trout (reported in Table 1). However, [Mg^2+^] and [SO_4_^2−^] are much higher in the posterior intestine of fasted trout in seawater compared with those in freshwater (Shehadeh and Gordon, 1969). Of particular note, osmolality of the luminal fluids in fed freshwater trout remains consistently higher than that in saline or plasma (Table 1). The presence of this *in vivo* mucosal-to-serosal osmotic gradient is important; fluid absorption by the gut *in vitro* is usually assessed under unrealistic conditions where mucosal and serosal media are isosmotic (Genz et al., 2011). If we wish to use *in vitro* protocols to accurately assess the transport of substances (particularly those for which solvent drag – see Glossary – is important), it will be necessary to ensure the appropriate osmolality of the mucosal medium *in vitro*.

### Mimicking the *in vivo* environment during *in vitro/ex vivo* experiments – a double-edged sword

Mimicking the variety of *in vivo* ionic, pH, [HCO_3_^−^] and ammonia conditions (Table 1) in *in vitro* experiments will involve the use of carefully designed buffered saline; obtaining the correct conditions of oxygen tension and the microbiome poses additional issues. In this section, we discuss potential approaches to address these challenges.

### Oxygen challenge

The most egregious deviation from natural conditions in almost all *in vitro* studies to date has been the use of high *P*_O_2__ (150 to >700 Torr) in the luminal fluid (see Table 1, and references therein). In humans and other vertebrates, the chyme is essentially anoxic, with a steep *P*_O_2__ gradient across the mucosal epithelium, such that the tip of the villi are nearly anoxic (reviewed by Espey, 2013; Ward et al., 2014; Singhal and Shah, 2020). With an anoxic lumen, oxygen delivery through the blood to the intestine is crucial for its optimal function. Oxygenated blood perfuses from the serosal side of the epithelium and is distributed to the mucosal folds, but this distribution is highly dynamic, depending on physiological state; consequently, the oxygen supply to the mucosa fluctuates widely (Taylor and Grosell, 2009; Seth et al., 2011). During fasting, oxygen supply does not meet the tissue demand, rendering the tip of the villi hypoxic, a state commonly referred to as ‘physiological hypoxia’. Only recently has luminal fluid *P*_O_2__ been measured *in vivo* in fish (Jung et al., 2020, 2022); it is less than 1 Torr in all gut sections in both fed and fasted animals (Table 1).

The main approach to oxygenating the tissue during *ex vivo* studies using techniques such as gut sacs and Ussing chambers is to gas the saline with 99.7% O_2_ (Wood and Grosell, 2012), 95% O_2_ (Bakke-McKellep et al., 2000) or 21% O_2_ (i.e. air levels; Sundell et al., 2003). In Ussing chamber studies and two-compartment *in vitro* cell culture models (see Glossary), both the mucosal and serosal side of the epithelium are usually gassed with the same gas mixture. However, N_2_ (with or without elevated *P*_CO_2__) could easily be used to create an anoxic environment on the mucosal side *in vitro* while oxygen is provided from the serosal side. It is very likely that high values of *P*_O_2__ on the luminal side of the epithelium (which have been used in virtually all *in vitro* investigations to date) result in severe oxidative stress for both the enterocytes and the resident microbial community, with unknown pathological effects on function.

### Microbiota challenge

As for other animals, the GI microbiome of fish plays a vital part in their health, nutrition and physiology (Xia et al., 2022). However, researchers have generally ignored the contribution of the microbiome because of the complex nature of the GI microbial ecosystem, which creates technical challenges that impede experimentation. Our resulting lack of knowledge regarding the intricate interactions between the microbiome and the host (and their consequences) prevents us from understanding the multifaceted physiology of the GI tract. In fact, the fish host plays a crucial role in shaping its GI microbiome through various physiological processes, including immune responses, gut motility and the secretion of mucus and antimicrobial peptides (e.g. Wiles et al., 2016; Maritan et al., 2024). These host–microbe interactions are essential for maintaining a balanced microbial community and ensuring proper gut function. However, *in vitro* and *ex vivo* models often lack these dynamic interactions (Wiles et al., 2016; Stagaman et al., 2017; Butt and Volkoff, 2019). In the following subsections, we discuss these interactions and how they may be captured in *in vitro* and *ex vivo* work.

### Microbial culturing

The elaborate network of bacteria, archaea, fungi and viruses that make up the GI microbiome are notorious for being difficult to isolate and culture using standard laboratory methods (e.g. Lau et al., 2016). Indeed, many of these microorganisms, especially obligate anaerobes and other fastidious species that require specific growth conditions, often fail to grow using standard culturing techniques. For example, the specific nutrient requirements and growth factors necessary for culturing many microbial species are not fully understood, impeding efforts to replicate the native microbiome accurately, although progress is being made (e.g. Lagier et al., 2018; Tao et al., 2023; Średnicka et al., 2023). Furthermore, the interactions among different microbial species in the gut are context dependent, involving symbiotic relationships such as mutualism, commensalism and competition (Pearce et al., 2018; Li et al., 2019). These relationships are difficult to replicate outside the host environment. As a result, the microbial communities cultured and then applied to experimental models may not accurately represent the communities in the fish gut, leading to biased interpretations and conclusions. Indeed, traditional culturing techniques often favour fast-growing, easy-to-culture species, and application of these specific bacteria to intestinal tissues and models results in an incomplete representation of the microbial diversity present in the fish GI tract (reviewed by Li et al., 2019; additional examples of monocultures and their impacts on non-fish models can be found in Pearce et al., 2018).

Advances in culturing techniques, such as the development of co-culture systems and the use of specialised growth media, have improved the ability to culture a broader range of gut microbes (Yadav et al., 2023). However, these methods are not yet capable of replicating the full diversity of the fish GI microbiome. Continued innovation in culturing techniques, including high-throughput culturing methods, ‘culturomics’ (see Glossary) and the use of gut-mimicking bioreactors, could help overcome these limitations so that the entirety of the community can be selectively applied to tissues for study.

There are further complicating factors in accurately representing the fish gut microbiome *in vitro*. The fish GI microbiome is significantly influenced by the surrounding aquatic environment, including variables such as water temperature and salinity (e.g. Legrand et al., 2020), and diet is another critical influencing factor (e.g. Butt and Volkoff, 2019). Even when complex mixtures of microorganisms are directly applied to *in vitro* models, the resulting communities are not representative of the initial communities; this is probably due to challenges in replicating biological conditions found in living GI systems. Further, microbial communities in the fish GI tract are not static; they continually adapt and evolve in response to environmental pressures and host interactions. This rapid adaptation and evolution is crucial for maintaining a balanced microbiome that can effectively respond to changes in the environment or host health.

### Microbe–mucus–epithelial interactions

The microbiota is highly beneficial for host health and produces metabolites that promote and improve health in general and intestinal health in particular. However, if homeostasis is threatened, the same microbiota can turn against the host and cause disease (Ghosh et al., 2021). The intricate balance between health and disease is regulated by the intestinal barriers, including the extrinsic secreted mucus barrier, the intrinsic epithelial barrier and the immune barrier known as the ‘gut-associated lymphoid system’ (see Glossary; Sundh and Sundell, 2015). Within the gut, mucus is continuously secreted, thereby pushing luminal bacteria away from the epithelium towards the lumen, where they can utilise the mucins in the mucus (and undigested biomolecules) as an energy source (Padra et al., 2017). If the mucus layer is compromised, the microbiota can attach to the epithelium, interact with the enterocytes, initiate translocation and stimulate an inflammatory response that weakens epithelial barrier function (Turner, 2009). Thus, mimicking the *in vivo* environment that includes endogenous microbiota will require an intact extrinsic mucus layer to prevent bacteria from interacting with the epithelium. However, preparation of tissues for *ex vivo* experiments (e.g. Ussing chamber) can cause mechanical damage that disrupts the normal status of the mucus layer, and *in vitro* preparations may lack mucus-secreting goblet cells. Thus, when preparing tissues for *ex vivo* studies, care should be taken to keep the protective mucus layer intact. In the future, the development of teleost intestinal organoids containing functional goblet cells could help to ensure that *in vitro* studies better reflect *in vivo* conditions.

### Microbial metabolome

The health benefits of the microbiota are mainly provided by the metabolome it produces in the lumen (reviewed by Hou et al., 2022; Ghosh et al., 2021). In humans, there are thousands of microbial metabolites, of which a few have been connected to beneficial intestinal function; these include short-chain fatty acids, indole derivates, bile acid metabolites, conjugated fatty acids, polyamines and polyphenolic derivatives (Ghosh et al., 2021). Thus, one approach to capturing the influence of the microbiota in an *in vitro* system may be to mimic the metabolome rather than the microbiota.

### *In vitro* models and development of microfluidic platforms

One of the principles of the 3Rs (Reduce, Refine and Replace; see Glossary) is to develop *in vitro* systems that aim to minimise the use of animals in physiology and toxicology studies. Complex tissues such as the gut are difficult to replicate, but significant progress has been made in recent years, particularly in mammalian gut research, where gut-on-chip devices have been specifically designed to mimic the physical conditions of the gut. The physical properties of the piscine gut are yet to be fully described, but when combined with the correct saline to mimic the chyme, oxygen gradients and microbiomes (or associated metabolites), an *in vitro* replacement that mimics the *in vivo* situation may be possible. Here, we discuss the current range of culture techniques that may, in the future, allow us to recapitulate the *in vivo* conditions in an *in vitro* system.

### Fish intestinal primary cell and immortalised cell cultures

Langan et al. (2018a) have described a protocol for gut epithelia cell isolation and primary cell culture for the different regions (pyloric, anterior, mid and posterior) of the rainbow trout gut. Of course, a primary cell culture system still requires donor fish, and within the 3Rs framework it is termed a ‘partial replacement’ for the use of live animals. Furthermore, care has to be taken in the culture conditions. Intestinal cells perform best at 21°C – compared with 15–18°C for rainbow trout gill primary cultures (Stott et al., 2015; Schnell et al., 2016) – and cells from pyloric caecae require MEM media (used in mammalian intestinal cell cultures), whereas the other regions require L-15 media, indicating that the former region requires different ratios of amino acids for sustenance (Bols et al., 1994b). Cells, when cultured on inserts, form epithelia with transepithelial resistance measurements of 60–90 Ω cm^2^, not too dissimilar from that measured in other salmonids (80–150 Ω cm^2^; Sundell and Sundh, 2012). Morphologically, the *in vitro* epithelium resembles intact intestinal cells, and it exhibits chemical biotransformation properties (cytochrome P450 activity) in the presence of organic chemicals and pharmaceuticals (Langan et al., 2018b,c). The versatility of this gut primary cell culture system has great potential that has yet to be fully utilised. Preparations from other species would allow for comparative studies, and growth of the epithelia on inserts enables the manipulation of culture conditions to mimic those found *in vivo*.

In addition, a rainbow trout gut cell line, RTgutGC was established by Kawano et al. (2011), and has been predominantly used in the replacement of fish in ecotoxicology experiments. The RTgutGC cell line, similar to the RTgill-W1 cell line (Bols et al., 1994a), has been suggested as a replacement for fish in acute toxicity studies (Schug et al., 2020) and in evaluating the toxicity of novel compounds (e.g. tyre tread particle toxicity; Dudefoi et al., 2024). These cells have also been used as an intestinal barrier model for nanoparticle uptake (Minghetti and Schirmer, 2016; Opršal et al., 2021). They can be cultured on membrane supports and show polarisation, a reasonable transepithelial resistance and apical localisation of the tight-junction protein ZO-1 (Minghetti et al., 2017). Growth of these cells with rainbow trout fibroblasts creates an intestinal–mesenchymal interface that is more representative of the *in vivo* gut epithelium than if the cells are grown on their own. This has been used for electrical impedance measurements (Drieschner et al., 2019a).

### Gut-on-chip devices

The two-compartment epithelial barrier model allows for independent control of luminal and serosal chamber conditions, including factors such as the microbiome and region-specific ionic composition and *P*_O_2__ gradients (Table 1). However, it does not recapitulate the dynamic environment of the gut, where, for example, fluid flows over the cells and muscle contractions cause peristalsis. Advancements in mammalian gut-on-chip technologies are starting to engineer devices that mimic this dynamic environment (Kim and Sung, 2024), and these issues are beginning to be addressed in the fish research community. A RTgutGC gut-on-chip device has been described by Driescher et al. (2019b), in which RTgutGC cells are co-cultured on ultrathin (500 nm) silicon nitride (Si_3_N_4_) membranes with a gut fibroblast cell line (RTgutF). An open fluidic circuit allows for fluid flow over the cells, and if similar flow conditions are applied to a mammalian intestinal cell line (Caco2 cells), the morphology of the cells alters such that they better represent columnar cells and folds that mimic the intact intestinal epithelium (Kim et al., 2012). Furthermore, stimulation of mechanosensory channels (i.e. Piezo1) in response to movement *in vivo* stimulates the goblet cells to release their contents into the lumen (Xu et al., 2021).

### *In vitro* gut microbiota

**Fig. 2.**
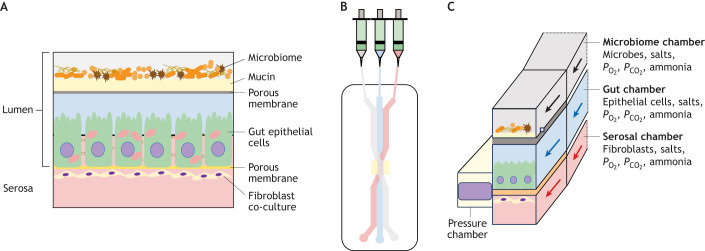
**Conceptual illustrations of a microfluidic teleost gut-on-chip.** (A) The top chamber contains the fish GI microbiome grown on a porous membrane covered in mucin, to allow the perfusion of metabolites into the middle chamber, which contains the gut epithelial cells grown on a second membrane. This forms the lumen. In the bottom (serosal) chamber, a layer of companion cells (e.g. fibroblasts) is grown on the other side of the membrane supporting the gut cells. (B) A representation of a microfluidic chip with the three separate chambers: the microbiome (grey channel), gut chamber (blue channel) and serosal chamber (pink channel). Each channel is connected to a syringe pump providing bespoke fluid to each chamber. The gut chamber is connected to a pressure chamber (yellow), which is designed to mimic the pressure changes associated with peristalsis. (C) A cross-section of the three chambers, with the pressure chamber designed to mimic the natural movement of the epithelium and a list of the chamber contents to the right. Based on the design by Marrero et al. (2021).

One of the major challenges of *in vitro* gut research will be the recapitulation of the gut microbiome. In an attempt to address this in a mammalian cell culture system, Kim et al. (2012) have demonstrated the co-culture of Caco-2 cells with the intestinal bacterium *Lactobacillus rhamnosus*, and this system has been further developed using gut-on-chip devices as a major tool to better understand microbiota interactions with the gut epithelium (e.g. Kim et al., 2015, 2019; Shah et al., 2016; Calatayud et al., 2019). The chip developed by Shah et al. (2016) is a modular microfluidic device that allows for the partitioning of intestinal microbes and Caco-2 cell cultures; specific conditions can be modified within the human cell and microbe chambers, and integrated sensors monitor *P*_O_2__ in the microchambers and the integrity of the membrane under experimental conditions. Using this system, it has been shown independently that incorporation of dynamic flow into the culture environment induces shear stress that is necessary for morphological changes and polarisation of cell cultures (Fois et al., 2021). Further developments in organoid cell cultures make use of the extracellular matrix as a cell substrate; these systems provide epithelia that better represent the tissues and provide high-throughput platforms (Morelli et al., 2023). Furthermore, Jalili-Firoozinezhad et al. (2019) have developed a method whereby a complex human gut microbiome can be cultured in an anaerobic intestine-on-a-chip, and we are seeing the development of multi-organ-on-chip devices (Ingber, 2022); such devices allow for the analysis of *in vitro* tissue–tissue interactions. This may offer a way forward for *in vitro* studies on fish intestinal function; a potential design for a microfluidic chip consisting of the fish gut epithelium as well as the microbiome is depicted in Fig. 2.

## Conclusion

Accurately recapitulating the environments of the different segments of the GI tract from various species of fish will require considerable knowledge of *in vivo* conditions*. In vitro* techniques should enable these conditions to be manipulated; however, almost all *ex vivo*/*in vitro* fish gut research to date has not carefully considered the unique gut chemistry, oxygen, carbon dioxide and ammonia gradients, physical environment or influence of microbiota and their metabolites. Until this can be achieved, reproducibility and interpretation of the results will require careful reporting of the culture conditions employed. In the future, the applicability of organ-on-chip devices is likely to be restricted to only a few species and is most likely to be used in aquaculture research to help improve productivity. Such investigations may focus on factors that influence the fish GI tract microbiome, nutrient uptake rates and dietary drug delivery. Incorporating realistic GI conditions in *in vitro*/*ex vivo* research has the potential to greatly improve our knowledge on basic fish gut physiology; for this to happen, we need greater focus on reporting species-specific luminal chemistries and microbial assemblages and an investment in developing fish-specific microphysiological platforms.
